# Supplementary material for: Large inversions in Lake Malawi cichlids are associated with habitat, lineage, and sex determination
Source: eLife. 2026 Nov 6;14:RP104923. doi: 10.7554/eLife.104923 (PMC13743080; doi:10.7554/eLife.104923)
Supplement: Supplementary file 13 [file elife-104923-supp4.docx]

| **Supplementary File 4** |  |  |  |  |
| --- | --- | --- | --- | --- |
| **Chromosome** | **Breakpoint 1 Interval Start** | **Breakpoint 1 Interval End** | **Breakpoint 2 Interval Start** | **Breakpoint 2 Interval End** |
| 2 | 19751534 | 19845509 | 43556655 | 43559056 |
| 9 | 14912159 | 14966448 | 32492000 | 32996230 |
| 10 | 11459086 | 11818098 | 29881276 | 29902559 |
| 11a | 8281988 | 8303032 | 18425177 | 18425215 |
| 11b | 18425177 | 18425215 | 30372599 | 30387602 |
| 13 | 2398108 | 2412755 | 23074431 | 23124401 |
| 20a | 19616377 | 19623494 | 29682528 | 29698815 |
| 20b | 29682528 | 29698815 | 33170004 | 33254382 |
