# Supplementary material for: Large inversions in Lake Malawi cichlids are associated with habitat, lineage, and sex determination
Source: eLife. 2026 Nov 6;14:RP104923. doi: 10.7554/eLife.104923 (PMC13743080; doi:10.7554/eLife.104923)
Supplement: Peer review history

# Editor report: eLife Assessment

**DOI:** 10.7554/eLife.104923.3.sa0

**Authors:** Virginie Courtier-Orgogozo

**Affiliations:** CNRS - Universite Paris Cite France

**Keywords:** Compelling, Important

Using several hundreds of samples and cutting-edge genomic methods, including BioNano, PacBio, HiFi, and advanced bioinformatic pipelines, the authors identify six large chromosomal inversions segregating in over 100 species of Lake Malawi cichlids. This **important** study provides **compelling** evidence for the presence of these six inversions, their differential distribution among populations, and the association of chromosome 10 inversion with a sex-determination locus. This work also provides a starting point for further investigating the role of these inversions with respect to local adaptation, speciation, sex determination, hybridization, and ILS in cichlids, which represent ~5% of the extant vertebrate species and are one of the most prominent examples of adaptive radiations.

# Referee report: Reviewer #2 (Public review)

**DOI:** 10.7554/eLife.104923.3.sa1

Summary:

Chromosomal inversions have been predicted to play a role in adaptive evolution and speciation because of their ability to "lock" together adaptive alleles in genomic regions of low recombination. In this study, the authors use a combination of cutting-edge genomic methods, including BioNano and PacBio HiFi sequencing, to identify six large chromosomal inversions segregating in over 100 species of Lake Malawi cichlids, a classic example of adaptive radiation and rapid speciation. By examining the frequencies of these inversions present in species from six different linages, the authors show that there is an association between the presence of specific inversions with specific lineages/habitats. Using a combination of phylogenetic analyses and sequencing data, they demonstrate that three of the inversions have been introduced to one lineage via hybridization. Finally, genotyping of laboratory crosses suggests that two inversions are associated with XY sex determination systems in a subset of species. The data add to a growing number of systems in which inversions have been associated with adaptation to divergent environments. However, like most of the other recent studies in the field, this study does not go beyond describing the presence of the inversions to demonstrate that the inversions are under sexual or natural selection or that they contribute to adaptation or speciation in this system.

Strengths:

All analyses are very well done, and the conclusions about the presence of the six inversions in Lake Malawi cichlids, the frequencies of the inversions in different species, and the presence of three inversions in the benthic lineages due to hybridization are well-supported. Genotyping of 48 individuals resulting from laboratory crosses provides strong support that the chromosome 10 inversion is associated with a sex-determination locus.

Weaknesses:

The evidence supporting a role for the chromosome 11 inversion is based on relatively few individuals and therefore remains suggestive. The authors are mostly cautious in their interpretations of the data, although there are places where the language is imprecise and therefore a little misleading.

# Author comment: Author response

**DOI:** 10.7554/eLife.104923.3.sa2

**Authors:** Nikesh M Kumar, Taylor L Cooper, Thomas Kocher, Jeffrey Todd Streelman, Patrick T McGrath

**Affiliations:** Georgia Institute of Technology Atlanta United States; Georgia Institute of Technology Atlanta United States; University of Maryland Maryland United States; Georgia Institute of Technology Atlanta United States; Georgia Institute of Technology Atlanta United States

The following is the authors’ response to the original reviews

We thank the reviewers for the careful review of our manuscript. Overall, they were positive about our use of cutting-edge methods to identify six inversions segregating in Lake Malawi. Their distribution in ~100 species of Lake Malawi species demonstrated that they were differentially segregating in different ecogroups/habitats and could potentially play a role in local adaptation, speciation, and sex determination. Reviewers were positive about our finding that the chromosome 10 inversion was associated with sex-determination in a deep benthic species and its potential role in regulating traits under sexual selection. They agree that this work is an important starting point in understanding the role of these inversions in the amazing phenotypic diversity found in the Lake Malawi cichlid flock.

There were two main criticisms that were made which we summarize:

(1) Lack of clarity. It was noted that the writing could be improved to make many technical points clearer. Additionally, certain discussion topics were not included that should be.

We will rewrite the text and add additional figures and tables to address the issues that were brought up in a point-by-point response. We will improve/include (1) the nomenclature to understand the inversions in different lineages, (2) improved descriptions for various genomic approaches, (3) a figure to document the samples and technologies used for each ecogroup, and (4) integration of LR sequences to identify inversion breakpoints to the finest resolution possible.

(2) We overstate the role that selection plays in the spread of these inversions and neglect other evolutionary processes that could be responsible for their spread.

We agree with the overarching point. We did not show that selection is involved in the spread of these inversions and other forces can be at play. Additionally, there were concerns with our model that the inversions introgressed from a *Diplotaxodon* ancestor into *benthic* ancestors and incomplete lineage sorting or balancing selection (via sex determination) could be at play. Overall, we agree with the reviewers with the following caveats. 1. Our analysis of the genetic distance between *Diplotaxodons* and *benthic* species in the inverted regions is more consistent with their spread through introgression versus incomplete lineage sorting or balancing selection. 2. Further the role of these inversions is likely different in different species. For example, the inversion of 10 and 11 play a role in sex determination in some species but not others and the potential pressures acting on the inverted and non-inverted haplotypes will be very different. These are very interesting and important questions booth for understanding the adaptive radiations in Lake Malawi and in general, and we are actively studying crosses to understand the role of these inversions in phenotypic variation between two species. We will modify the text to make all of these points clearer.

**Public Reviews:**

**Reviewer #1 (Public review):**

Summary:

Using high-quality genomic data (long-reads, optical maps, short-reads) and advanced bioinformatic analysis, the authors aimed to document chromosomal rearrangements across a recent radiation (Lake Malawi Cichlids). Working on 11 species, they achieved a high-resolution inversion detection and then investigated how inversions are distributed within populations (using a complementary dataset of short-reads), associated with sex, and shared or fixed among lineages. The history and ancestry of the inversions is also explored.

On one hand, I am very enthusiastic about the global finding (many inversions well-characterized in a highly diverse group!) and impressed by the amount of work put into this study. On the other hand, I have struggled so much to read the manuscript that I am unsure about how much the data supports some claims. I'm afraid most readers may feel the same and really need a deep reorganisation of the text, figures, and tables. I reckon this is difficult given the complexity brought by different inversions/different species/different datasets but it is highly needed to make this study accessible.

The methods of comparing optical maps, and looking at inversions at macro-evolutionary scales can be useful for the community. For cichlids, it is a first assessment that will allow further tests about the role of inversions in speciation and ecological specialisation. However, the current version of the manuscript is hardly accessible to non-specialists and the methods are not fully reproducible.

Strengths:

(1) Evidence for the presence of inversion is well-supported by optical mapping (very nice analysis and figure!).

(2) The link between sex determination and inversion in chr 10 in one species is very clearly demonstrated by the proportion in each sex and additional crosses. This section is also the easiest to read in the manuscript and I recommend trying to rewrite other result sections in the same way.

(3) A new high-quality reference genome is provided for Metriaclima zebra (and possibly other assemblies? - unclear).

(4) The sample size is great (31 individuals with optical maps if I understand well?).

(5) Ancestry at those inversions is explored with outgroups.

(6) Polymorphism for all inversions is quantified using a complementary dataset.

Weaknesses:

(1) Lack of clarity in the paper: As it currently reads, it is very hard to follow the different species, ecotypes, samples, inversions, etc. It would be useful to provide a phylogeny explicitly positioning the samples used for assembly and the habitat preference. Then the text would benefit from being organised either by variant or by subgroups rather than by successive steps of analysis.

We have extensively rewritten the paper to improve the clarity. With respect to this point, we moved Figure 6 to Figure 1, which places the phylogeny of Lake Malawi cichlids at the beginning of the paper. We incorporated information about samples/technologies by ecogroup into this figure to help the reader gain an overview of the technologies involved. We added information about habitat for each ecogroup as well. While we considered a change to the text organization suggested here, we thought it was clearer to keep the original headings.

(2) Lack of information for reproducibility: I couldn't find clearly the filters and parameters used for the different genomic analyses for example. This is just one example and I think the methods need to be re-worked to be reproducible. Including the codes inside the methods makes it hard to follow, so why not put the scripts in an indexed repository?

We now provide a link to a github repository (https://github.com/ptmcgrat/CichlidSRSequencing/tree/Kumar_eLife) containing the scripts used for the major analysis in the paper. Because our data is behind a secure Dropbox account, readers will not be able to run the analysis, however, they can see the exact programs, filters, and parameters used for manuscript embedded within each script.

(3) Further confirmation of inversions and their breakpoints would be valuable. I don't understand why the long-reads (that were available and used for genome assembly) were not also used for SV detection and breakpoint refinement.

We did use long reads to confirm the presence of the inversions by creating five new genome assemblies from the PacBio HiFi reads: two additional *Metriaclima zebra* samples and three *Aulonocara* samples. Alignment of these five genomes to the MZ_GT3 reference is shown in Figures S2 – S7. These genome assemblies were also used to identify the breakpoints of the inversions. However, because of the extensive amount of repetitive DNA at the breakpoints (which is known to be important for the formation of large inversions), our ability to resolve the breakpoints was limited.

(4) Lack of statistical testing for the hypothesis of introgression: Although cichlids are known for high levels of hybridization, inversions can also remain balanced for a long time. what could allow us to differentiate introgression from incomplete lineage sorting?

The coalescent time between the inversions between Diplotaxodons and benthics should allow us to distinguish these two mechanisms. Our finding that the genetic distance, which is related to coalescent time, is closer within the inversions than the whole genome is supportive of introgression. However, we did not perform any simulations or statistical tests. We make it clearer in the text that incomplete lineage sorting remains a possible mechanism for the distribution of inversions within these ecogroups.

(5) The sample size is unclear: possibly 31 for Bionano, 297 for short-reads, how many for long-reads or assemblies? How is this sample size split across species? This would deserve a table.

We have included this information in the new Figure 1.

(6) Short read combines several datasets but batch effect is not tested.

We do not test for batch effect. However, we do note that all of the datasets were analyzed by the same pipeline starting from alignment so batch effects would be restricted to aspects of the reads themselves. Additionally, samples from the different data sets clustered as expected by lineage and inferred inversion, so for these purposes unlikely to have affected analysis.

(7) It is unclear how ancestry is determined because the synteny with outgroups is not shown.

Ancestry analysis was determined using the genome alignments of two outgroups from outside of Lake Malawi. This is shown in Figure S8.

(8) The level of polymorphism for the different inversions is difficult to interpret because it is unclear whether replicated are different species within an eco-group or different individuals from the same species. How could it be that homozygous references are so spread across the PCA? I guess the species-specific polymorphism is stronger than the ancestral order but in such a case, wouldn't it be worth re-doing the PCa on a subset?

The genomic PCA plots reflect the evolutionary histories that are observed in the whole genome phylogenies. Because the distribution of the inverted alleles violate the species tree, they form separate clusters on the PCA plots that can be used to genotype specific species. We have also performed this analysis on benthics (utaka/shallow benthics/deep benthics) and the distribution matches the expectation.

**Reviewer #2 (Public review):**

Summary:

Chromosomal inversions have been predicted to play a role in adaptive evolution and speciation because of their ability to "lock" together adaptive alleles in genomic regions of low recombination. In this study, the authors use a combination of cutting-edge genomic methods, including BioNano and PacBio HiFi sequencing, to identify six large chromosomal inversions segregating in over 100 species of Lake Malawi cichlids, a classic example of adaptive radiation and rapid speciation. By examining the frequencies of these inversions present in species from six different linages, the authors show that there is an association between the presence of specific inversions with specific lineages/habitats. Using a combination of phylogenetic analyses and sequencing data, they demonstrate that three of the inversions have been introduced to one lineage via hybridization. Finally, genotyping of wild individuals as well as laboratory crosses suggests that three inversions are associated with XY sex determination systems in a subset of species. The data add to a growing number of systems in which inversions have been associated with adaptation to divergent environments. However, like most of the other recent studies in the field, this study does not go beyond describing the presence of the inversions to demonstrate that the inversions are under sexual or natural selection or that they contribute to adaptation or speciation in this system.

Strengths:

All analyses are very well done, and the conclusions about the presence of the six inversions in Lake Malawi cichlids, the frequencies of the inversions in different species, and the presence of three inversions in the benthic lineages due to hybridization are well-supported. Genotyping of 48 individuals resulting from laboratory crosses provides strong support that the chromosome 10 inversion is associated with a sex-determination locus.

Weaknesses:

The evidence supporting a role for the chromosome 11 inversion and the chromosome 9 inversion in sex determination is based on relatively few individuals and therefore remains suggestive. The authors are mostly cautious in their interpretations of the data. However, there are a few places where they state that the inversions are favored by selection, but they provide no evidence that this is the case and there is no consideration of alternative hypotheses (i.e. that the inversions might have been fixed via drift).

We have removed mention of chromosome 9’s potential role in sex determination from the paper. While our analysis of sex association with chromosome 11 was limited compared to our analysis of chromosome 10, it was still statistically significant, and we believe it should be left in the paper. The role of 11 (and 9 and 10) in sex determination was also demonstrated using an independent dataset by Blumer et al (https://doi.org/10.1101/2024.07.28.605452)

We agree that we did not properly consider alternative hypothesis in the original submission and have rewritten the Discussion substantially to consider various alternative hypothesis.

**Reviewer #3 (Public review):**

This is a very interesting paper bringing truly fascinating insight into the genomic processes underlying the famous adaptive radiation seen in cichlid fishes from Lake Malawi. The authors use structural and sequence information from species belonging to distinct ecotypic categories, representing subclades of the radiation, to document structural variation across the evolutionary tree, infer introgression of inversions among branches of the clade, and even suggest that certain rearrangements constitute new sex-determining loci. The insight is intriguing and is likely to make a substantial contribution to the field and to seed new hypotheses about the ecological processes and adaptive traits involved in this radiation.

I think the paper could be clarified in its prose, and that the discussion could be more informative regarding the putative roles of the inversions in adaptation to each ecotypic niche. Identifying key, large inversions shared in various ways across the different taxa is really a great step forward. However, the population genomics analysis requires further work to describe and decipher in a more systematic way the evolutionary forces at play and their consequences on the various inversions identified.

The model of evolution involving multiple inversions putatively linking together co-adapted "cassettes" could be better spelled out since it is not entirely clear how the existing theory on the recruitment of inversions in local adaptation (e.g. Kirkpatrick and Barton) operates on multiple unlinked inversions. How such loci correspond to distinct suites of integrated traits, or not, is not very easy to envision in the current state of the manuscript.

This is a very interesting point, and we agree creates complications for a simple model of local adaptation. We imagine though that the actual evolutionary history was much more complicated than a single Rhamphochromis-type species separating from a single Diplotaxodon-type species and could have occurred sequentially involving multiple species that are now extinct. A better understanding of the role each of these inversions play in phenotypic diversity could potentially help us determine if different inversions carry variation that could be linked to distinct habit differences. We have added a line to the discussion.

The role of one inversion in sex determination is apparent and truly intriguing. However, the implication of such locus on ecological adaptation is somewhat puzzling. Also, whether sex determination loci can flow across species via introgression seems quite important as a route to chromosomal sex determination, so this could be discussed further.

Another very interesting point. If the inversions are involved in ecological adaptation (an important caveat), then potentially the inverted and non-inverted haplotypes play dual roles in the Aulonocara animals with the inverted haplotype carrying adaptive alleles to deep water and the non-inverted haplotype carrying alleles resolving sexual conflict. We have broadened our discussion about their function at the origin including non-adaptive roles.

**Recommendations for the authors:**

**Reviewer #2 (Recommendations for the authors):**

Overall, the paper is well-written and clear. I do have a few suggestions for changes that would help the reader:

(1) Figure 1: the figure legend could be expanded here to help the reader; what are the blue and yellow lines? Why are there two lines for the GT3a assembly? And, I had to somehow read the legend a few times to understand that the top line is the UMD2a reference assembly, and the next line is the new Bionano map.

Fixed in what is now Figure 2

(2) Paragraph starting on line 133: you use the word "test" to refer to the Bionano analyses; it is not clear whether anything is being tested. Perhaps "analyse the maps" or just "map" would be more clear? Or more explanation?

The text has been modified to address this point

(3) L145-146: perhaps change "a single inversion" and "a double inversion" to "single inversions" and "double inversions".

The text has been modified to address this point

(4) L157: suppression of recombination in inversion heterozygotes is "textbook" material and perhaps does not need a reference. Or, you could reference an empirical paper that demonstrates this point. Though I love the Kirkpatrick and Barton paper, it certainly is not the correct reference for this point.

The Kirkpatrick reference was incorrectly included here. The correct reference was an empirical demonstration (Conte) that there were regions of suppressed recombination that have been observed in the location of the inversions. We have also moved this reference further up in the sentence to a more appropriate position

(5) L173: how do you know this is an assembly error and not polymorphism?

The text has been modified to address this point

(6) L277(?): "currently growing in the lab" is probably unnecessary.

The text has been modified to address this point

(7) L298: "the inversion on 10 acts as an XY sex determiner": the inversion itself is not the sex determination gene; rather, it is linked. I think it would be more precise, here and throughout the paper, to say that these inversions likely harbor the sex determination locus (for example, the wording on lines 369-370 is misleading).

We agree with the larger point that the inversion might not be causal for sex determination, however, it could still be causal through positional effects. We have modified the text to make it clear that it could also carry the causal locus (or loci).

(8) Figure 6: overall, this figure is very helpful! However, it contains several problematic statements. In no case do you have evidence that these inversions are "favored by selection"; such statements should be deleted. Also, in point 3, you state that inversions 9, 11, and 20 are transferred to benthic lineages, and then that these inversions are involved in sex determination. But, your data suggests that it is chromosomes 9, 10, and 11 that are linked to sex determination.

This figure is now Figure 1. We have remove these problematic statements.

(9) L356-360: I would move the references that are currently at the end of the sentence to line 357 after the statement about the previous work on hybridization. Otherwise, it reads as if these previous papers demonstrated what you have demonstrated in your work.

The text has been modified to address this point

(10) Overall, the discussion focuses completely on adaptive explanations for your results, and I would like to see at least an acknowledgement that drift could also be involved unless you have additional data to support adaptive explanations.

We have rewritten the text to account for the possibility of drift (line 404 and 405).

**Reviewer #3 (Recommendations for the authors):**

The paper utilizes heterogeneous datasets coming from different sources, and it is not always clear which specimens were used to generate structural information (bionano) or sequence information. A diagram summarizing the sequence data, methodologies, and research questions would be beneficial for the reader to navigate in this paper.

Much of this information has been added to what is now Figure 1. All of this data is also found in Table S2.

The authors performed genome alignments to analyze and homologize inversion, but this process is not clearly described. For the PCA, SNP information likely involves mapping onto a common reference genome. However, it is not clear how this was achieved given the different species and varying divergence times involved.

We now include a link to the github that contains the commands that were run. Because the overall level of sequence divergence between cichlid species is quite low (2*10^-3 – Milansky et al), mapping different species onto a common reference is commonly performed in Lake Malawi cichlids.

The introgression scenario is very intriguing but its role in local adaptation of the ecogroup types is not easy to understand. I understand this is still an outstanding question, but it is unclear how the directionality of introgressions was estimated. This can be substantiated using tree topology analysis, comparative estimates of sequence divergence, and accumulation of DNA insertions. The diagram does not clearly indicate which ones are polymorphic. In some cases, polymorphic inversions could result from the coexistence of native and introgressed haplotypes.

We agree that this analysis would be interesting but is beyond the scope of this paper.

The alternative model of introgression proposed in the cited preprint is interesting and should deserve a formal analysis here. The authors consider unclear what would drive "back" introgressions of non-inverted haplotypes, but this would depend on the selection regimes acting on the inversions themselves, which can include forms of balancing selection and a role for recessive lethals (heterozygote advantage). For instance, a standard haplotype could be favored if it shelters deleterious mutations carried by an inversion. Testing the introgression history over a wider range of branches and directions would provide further insights.

We agree that this analysis would be interesting but is beyond the scope of this paper.

The prose in the paper is occasionally muddled and somewhat unclear. Referring to chromosomes solely by their numbers (e.g.. "inversion on 11") complicates readability.

This is the standard way to refer to chromosomes in cichlids and we believe while it complicates readability, any other method would be inconsistent with other papers. Changes to nomenclature might improve the readability of this paper, but would make it more difficult to compare results for these chromosomes from other papers with what we have found.
